# Runs of Homozygosity Predict Inbreeding Depression Across Taxa: A Systematic Review and Meta‐Analysis

**DOI:** 10.1111/mec.70541

**Published:** 2026-09-01

**Authors:** Julia C. Clarke, Rebecca S. Taylor, Micheline Manseau

**Affiliations:** ^1^ On Contract With Science and Technology, Environment and Climate Change Canada Ottawa Ontario Canada; ^2^ Science and Technology, Environment and Climate Change Canada Ottawa Ontario Canada

**Keywords:** fitness, inbreeding, reproduction, runs of homozygosity, survival, wildlife conservation

## Abstract

Measuring inbreeding via runs of homozygosity (ROH) captures realized autozygosity and can infer inbreeding timing through ROH length. A growing body of literature links the proportion of the genome in ROH (F_ROH_) to fitness outcomes across taxa, yet systematic synthesis has been lacking. Here, we conduct a systematic review and meta‐analysis to quantify F_ROH_‐fitness associations, identify drivers of variation and derive conservation‐relevant recommendations. Narrative synthesis of 44 studies revealed that inbreeding depression operates through multiple interconnected pathways (survival, maternal effects, disease susceptibility, reproduction). Critically, purging cannot be relied upon to eliminate inbreeding depression as substantial fitness costs persist even in historically small populations. Meta‐analysis of 62 effect sizes revealed a significant negative association between genomic inbreeding and fitness across taxa (Fisher's z = −0.103, *r* = −0.10, *p* < 0.0001). Study group, whether wildlife, livestock or humans, explained 22.5% of variance, with wildlife showing strongest effects (6‐fold stronger than humans). Survival traits showed the greatest sensitivity to the effects of ROH (*r* = −0.22). Additionally, ROH detection methodology significantly influenced effect sizes: comprehensive approaches (all ROH lengths) detected stronger depression (*r* = −0.18) than long‐ROH‐only analyses (*r* = −0.08, *p* = 0.008), indicating cumulative genetic load matters. Overall, results indicate significant but variable fitness associations with ROH, with effect magnitude depending on biological context and methodological approach. Comprehensive ROH‐based approaches show promise as conservation monitoring tools, but limited wildlife studies, particularly for non‐mammalian taxa, highlight an urgent need for standardized protocols and expanded empirical research.

## 1 Inbreeding Depression and Runs of Homozygosity

Inbreeding depression, the reduction in fitness resulting from increased homozygosity, is a key threat to small and isolated wildlife populations. Monitoring inbreeding is therefore a conservation priority and has traditionally relied on pedigree‐based inbreeding coefficients (FPED), which require extensive long‐term demographic data that are rarely available for wild populations (Pemberton 2008). As genomic tools become more accessible, runs of homozygosity (ROH) and ROH‐based inbreeding coefficients (F_ROH_) derived from SNP arrays and whole‐genome sequencing have emerged as superior alternatives, capturing both recent and ancestral inbreeding history in ways that pedigree‐based measures cannot (Kardos et al. 2015). A growing body of research across study systems (predominantly livestock, wildlife and humans) is now examining the relationship between genomic inbreeding and fitness, yet how reliably F_ROH_ predicts fitness outcomes and under what biological and methodological conditions remains poorly understood. Here, we present a systematic literature review and meta‐analysis synthesizing methodological approaches, comparing findings across study systems and estimating a pooled effect size for the relationship between genomic inbreeding and fitness across wildlife, livestock and human populations.

## 2 Literature Review and Statistical Analysis

A systematic literature review was conducted following PRISMA guidelines to identify studies examining relationships between genomic inbreeding, measured as runs of homozygosity (ROH) and fitness outcomes (Figure S1). Database searches were performed in Web of Science and Google Scholar using search terms combining genomic inbreeding terminology (e.g., ‘runs of homozygosity,’ ‘ROH,’ ‘FROH,’ ‘genomic inbreeding’) with fitness‐related terms (e.g., ‘fitness,’ ‘survival,’ ‘reproduction,’ ‘inbreeding depression’). The full search terms and strategies for each database are provided in Tables S1 and S2. Studies were included if they: (1) used ROH‐based genomic inbreeding measures rather than pedigree‐based coefficients; (2) reported quantifiable relationships between F_ROH_ and fitness traits; and (3) provided sufficient statistical information for effect size extraction. Studies using only pedigree‐based inbreeding measures, microsatellite markers or any other non‐robust method of estimating genomic inbreeding were excluded. For the purposes of the narrative synthesis, studies were included if there was enough information to make inferences about fitness (e.g., observations, clinical health data from records, etc.), however, without extractable fitness correlations these studies were excluded from the meta‐analysis portion of the review.

Data were extracted into two linked databases (Data S1): a study‐level database capturing genomic methodology (species, sample size, sequencing platform, SNP density, ROH detection software and parameters) and an effect‐level database recording individual F_ROH_‐fitness relationships (trait category, effect size, sample size, statistical significance). Studies were categorized by study system (human, livestock, wildlife) and fitness traits were classified as survival/longevity, reproduction/fertility, morphology/growth, disease/immune function or production (livestock only). ROH detection parameters including minimum length thresholds and whether studies restricted analyses to long ROH (> 1 Mb) were recorded. This 1 Mb threshold was chosen based on recommendations from previous studies (Ceballos et al. 2018; Kyriazis et al. 2025).

Effect sizes were converted to Fisher's z transformation to enable meta‐analytic synthesis. Pearson and Spearman correlations were directly transformed using *z* = 0.5 × ln[(1 + *r*)/(1 − *r*)]. Standardized regression coefficients from simple regressions were treated as correlation approximations. Odds ratios were converted using *r* = ln(OR)/1.81 assuming standard logistic distributions. Standard errors were calculated as SE = 1/√(*n* − 3). Non‐significant effects were retained to minimize bias.

Meta‐analysis was conducted using the metafor package in R (version 4.5.2). Random‐effects models were fitted using restricted maximum likelihood (REML) estimation. Outliers were identified using standardized residuals (|*z*| > 3) and removed prior to analyses. Heterogeneity was assessed using *I* ^2^ and τ^2^ statistics. Mixed‐effects meta‐regression tested three moderators: study system (human, livestock, wildlife), fitness trait category and ROH detection threshold (whether studies used only ROH > 1 Mb versus including shorter segments). A combined model incorporating all significant moderators was fitted to estimate total variance explained. Publication bias was assessed using Egger's regression test and trim‐and‐fill analysis.

Because heterogeneity across studies was very high (*I* ^2^≈99%), a fixed‐effect model, which assumes all studies share one true effect size, was not appropriate. A random‐effects model instead allows the true effect to differ from study to study, better reflecting biological and methodological differences across taxonomic groups, trait categories and ROH detection approaches. We chose REML because it produces less biased estimates of between‐study variance (τ^2^) than standard maximum likelihood, especially with a moderate number of studies (k = 23). To help explain this heterogeneity, we ran moderator analyses using taxonomic group, trait category and ROH detection threshold as predictors, which together accounted for about 36.8% of the variation.

Non‐independence among effect sizes within studies was not explicitly modelled via a multilevel or robust variance estimation (RVE) structure, but this is unlikely to affect conclusions: τ^2^ already dominates total variance given *I* ^2^≈99%, leaving little room for clustering to alter results. With k = 23 studies contributing 62 effect sizes, several with only one or two, a multilevel model risks overfitting and unstable estimates while reducing power for moderator analyses. Ignoring clustering tends to underestimate standard errors, which would work against, not in favour of, our generally modest, non‐significant findings (e.g., pooled *r* ≈ −0.10).

## 3 Study Characteristics and Methodological Heterogeneity

Initial searches identified 224 potentially relevant studies. After a more critical full‐text review, 44 studies met final inclusion criteria (Figure S1). These 44 studies were included in the literature review and synthesis, while the final meta‐analysis included 62 effect sizes from 23 studies after excluding those whose effect sizes could not be converted to Fisher's z. Studies were dominated by livestock populations (*n* = 22), followed by wildlife (*n* = 13) and human studies (*n* = 10). A total of 92 fitness effect estimates were extracted from all studies with reproduction and fertility traits being reported most commonly (*n* = 28), followed by morphology and growth (*n* = 26), survival and longevity (*n* = 22), disease and immunity (*n* = 19) and production traits (*n* = 16).

Considerable variation was identified in ROH detection methodology. PLINK software was the most commonly used ROH‐calling software (*n* = 34 of 45 studies), though parameter settings were highly variable. SNP density ranged from 5 to 10,000 SNPs/Mb (median = 39 SNPs/Mb), which reflects the tendency for livestock studies to use 50 K SNP arrays versus wildlife and human studies tending towards whole‐genome sequencing or imputation from SNP arrays to higher densities. Notably, 44% of studies used SNP densities below the recommended 22 SNPs/Mb for reliable PLINK‐based ROH detection (Lavanchy and Goudet 2023). Minimum ROH length thresholds ranged from 100 Kb to 5 Mb, with 1–2 Mb being most common (mean = 1.2 Mb).

## 4 ROH Impact Fitness Across Studies

The reviewed studies documented inbreeding depression operating through multiple pathways, including direct effects on survival and longevity (Hasik et al. 2025; Huisman et al. 2016; Kardos et al. 2023; Niskanen et al. 2020; Stoffel et al. 2021b, 2021a; Townsend and Jamieson 2013), reduced fertility and reproductive success (Alshanbari and Aloraini 2025; Foster et al. 2026; Huisman et al. 2016; Kim et al. 2015; Laseca et al. 2022; Saura et al. 2015; Swinford 2023), increased disease susceptibility (Christofidou et al. 2015; Gates et al. 2025; Hasik et al. 2025; Mooney et al. 2021) and impaired growth (Belbin et al. 2017; Bérénos et al. 2016; Hasik et al. 2025; Matenchi et al. 2025; Reverter et al. 2017; Sumreddee et al. 2019). Studies separating inbreeding effects between maternal and offspring F_ROH_, demonstrate that maternal inbreeding can reduce offspring fitness independently of offspring inbreeding status (Bérénos et al. 2016; Stoffel et al. 2021a). Additionally, population‐wide patterns of ROH distribution influenced fitness outcomes: regions showing elevated homozygosity across many individuals (ROH islands) likely reflect positive selection rather than inbreeding depression (e.g., Colpitts et al. 2022). Studies examining ROH length classes found that longer segments (> 1–2 Mb) had stronger per‐segment effects on fitness than shorter segments (e.g., Kardos et al. 2018).

A random‐effects meta‐analysis revealed a significantly negative relationship between genomic inbreeding (F_ROH_) and fitness (*r* = −0.10, *p* < 0.0001; Figure S2). Three outliers were identified and removed based on their standardized residuals being greater than ±3. High heterogeneity (*I* ^2^ = 98.71) indicated considerable variation in effect sizes across studies, validating a need for moderator analyses.

## 5 Effects of Study Systems, Traits and ROH Lengths

**FIGURE 1:**
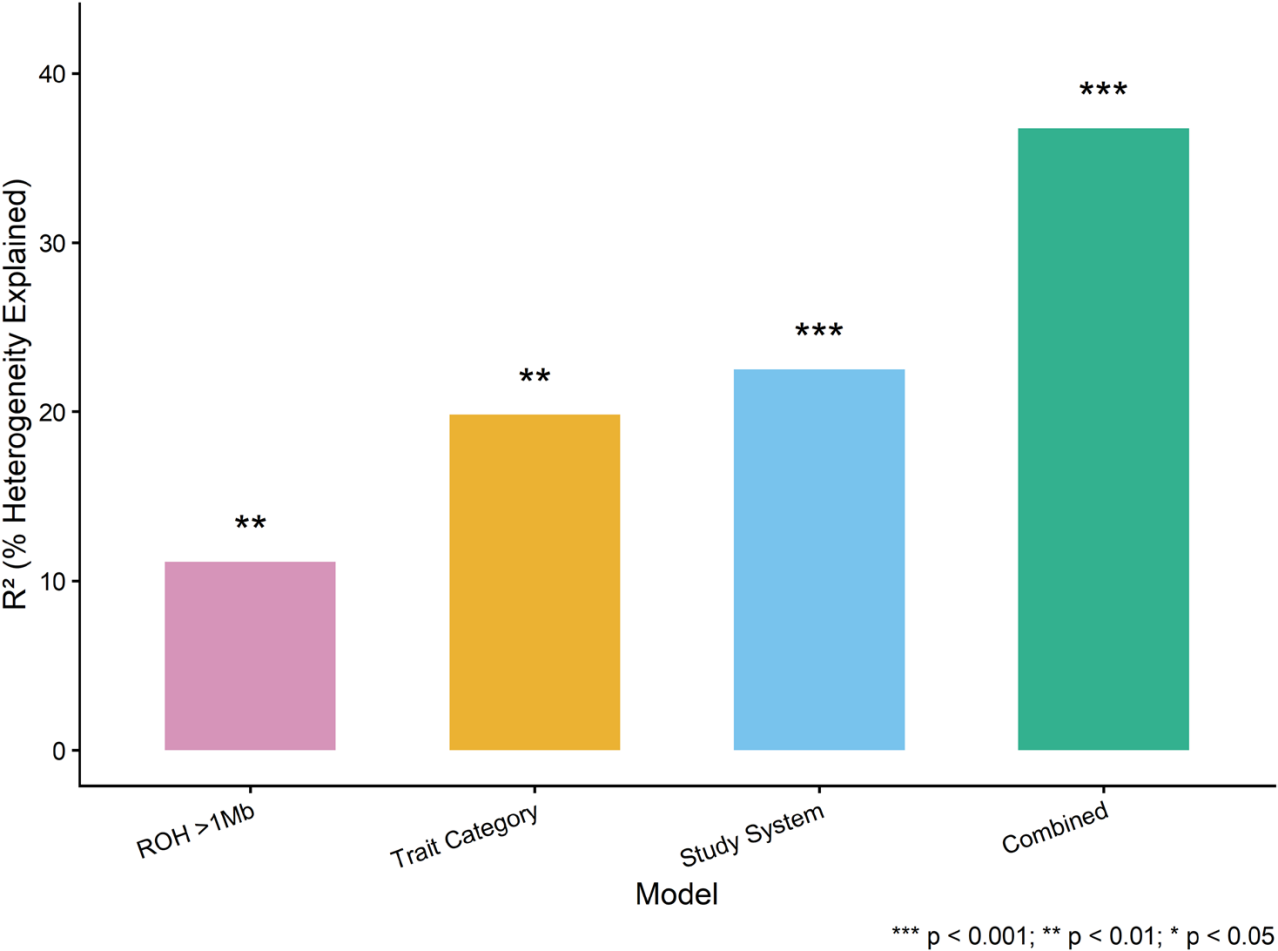
The percentage of the heterogeneity in F_ROH_‐fitness relationships explained by ROH length threshold, fitness trait category, study system, as well as for a combined model incorporating all three.

**FIGURE 2:**
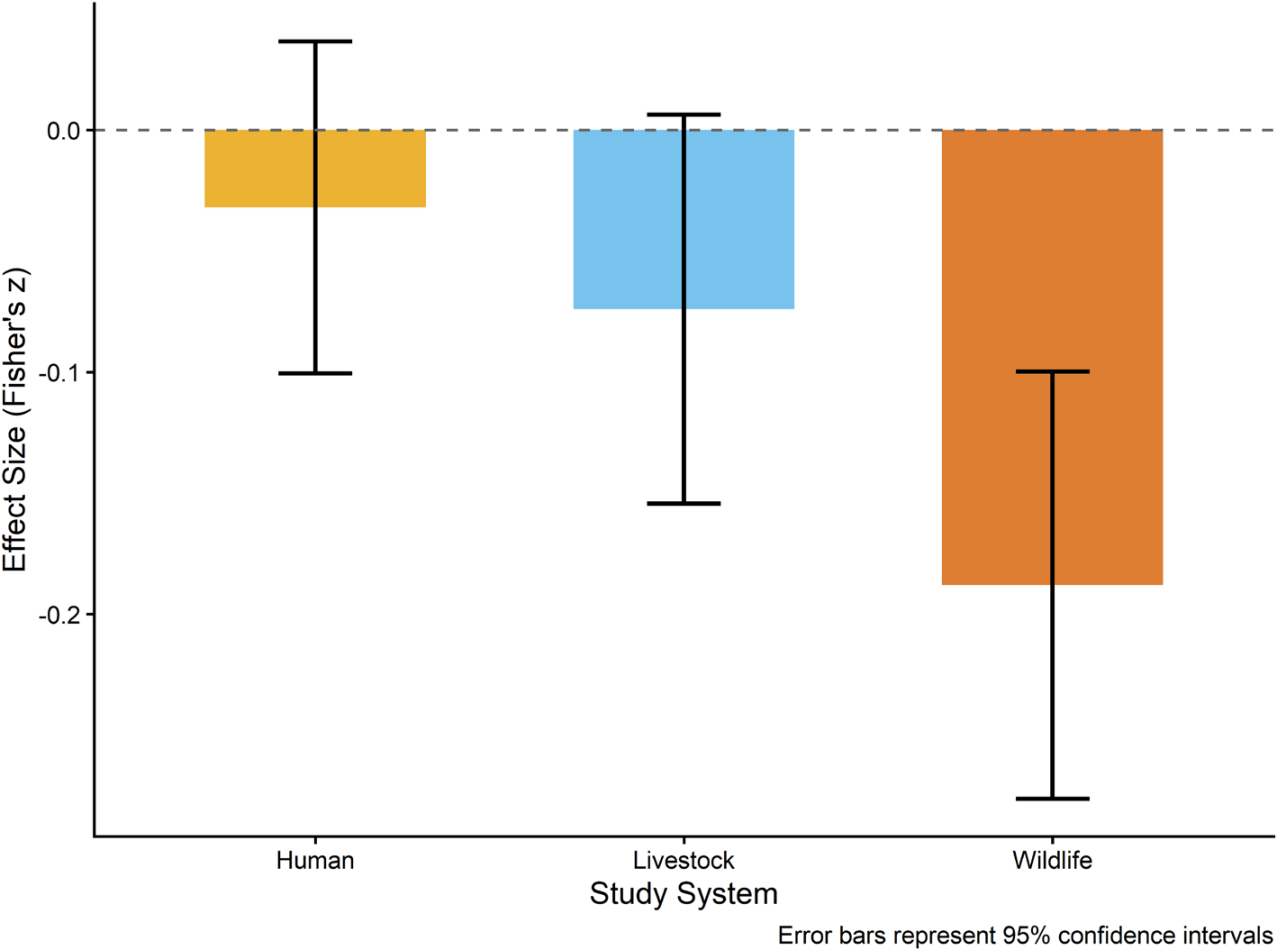
The effect sizes for the relationship between genomic inbreeding as measured with ROH and fitness in the three study systems: Human, livestock and wildlife.

**FIGURE 3:**
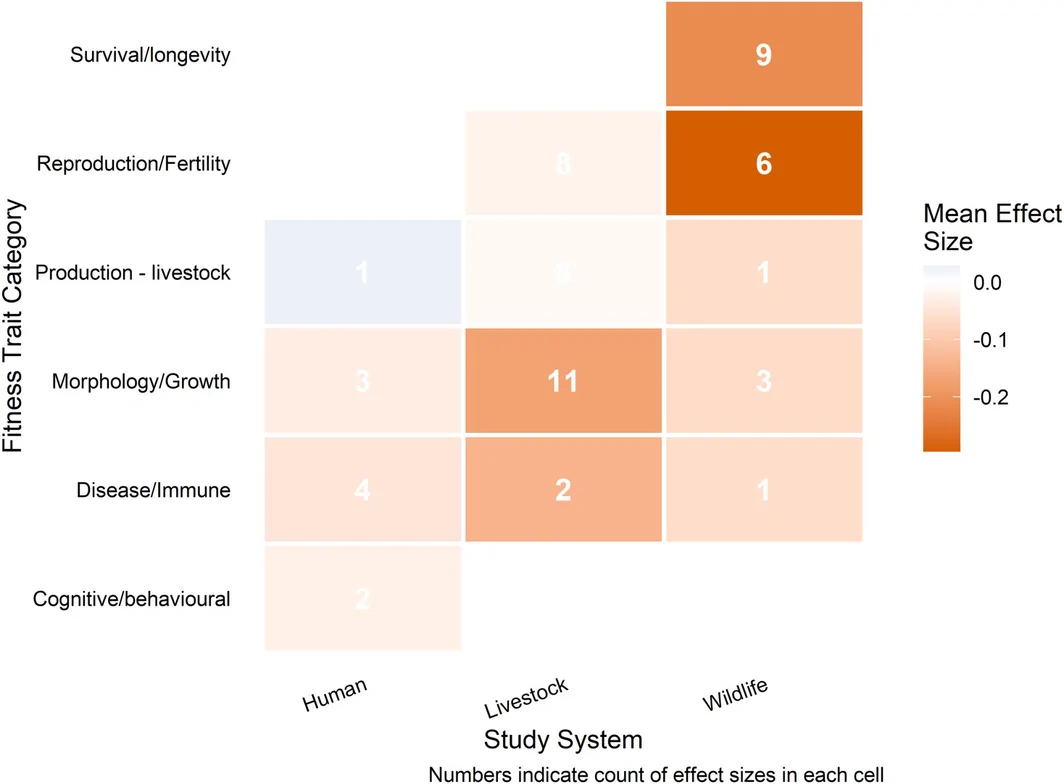
The mean effect size shown by fitness trait category and study system, with larger effect sizes in darker red. The counts of the effect sizes are included within each cell.

Three moderators (study system, fitness trait category and ROH length threshold) explained a significant amount of variation in F_ROH_‐fitness relationships. Study system was a significant predictor, explaining 22.5% of the variance (Figure 1), with wildlife populations showing the strongest inbreeding depression (*r* = −0.188; Figure 2). Fitness trait category was also a significant predictor, explaining 19.82% of the variance, with survival and longevity traits showing the strongest inbreeding depression (*r* = −0.217) followed by reproduction and fertility (*r* = −0.116), morphology and growth (*r* = −0.111) and disease and immunity (*r* = −0.085; Figure 3). Production traits in livestock showed the weakest effects (*r* = −0.011; Figure 3).

ROH detection threshold emerged as a novel methodological moderator, explaining 11.14% of variance (*p* = 0.008; Figure 1). Studies that restricted their analyses to only long ROH (≥ 1 Mb) detected weaker inbreeding depression (*r* = −0.078) compared to studies including shorter ROH segments (*r* = −0.181).

A combined model incorporating study system, trait category and ROH threshold explained 36.76% of heterogeneity (*p* < 0.0001; Figure 1). Study system and ROH threshold effects remained significant after mutual adjustment, while trait category effects became non‐significant when controlling for taxonomy, indicating study system as the primary biological moderator. An Egger's regression test identified funnel plot asymmetry, suggesting publication bias (Figure S3). A trim‐and‐fill analysis suggested a total of four missing studies, giving us an adjusted overall effect size of *r* = −0.083, which remained statistically significant.

## 6 ROH in Wildlife and Recommendations

Our meta‐analysis provides the first cross‐taxon quantification of the relationship between ROH‐based inbreeding estimates and fitness, demonstrating a significant and consistent negative association between genomic inbreeding (F_ROH_) and fitness across wildlife, livestock and human populations combined (*r* = −0.10, *p* < 0.00001), though effect magnitude varied considerably across studies. Wildlife populations showed consistently stronger inbreeding depression effects, approximately six times larger than those observed in humans (Figure 2), consistent with theory predicting stronger effects in small, fragmented populations facing intensifying habitat loss.

As genomic tools become increasingly accessible for non‐model species, studies examining the relationship between ROH and fitness have proliferated across taxa and study systems, creating an opportunity to quantify overall effects, identify consistent patterns and guide methodological standardization for conservation applications. Realizing this opportunity requires grappling with the considerable methodological heterogeneity identified here, which has direct consequences for cross‐study comparability and the detection of inbreeding depression. Studies using sparse SNP panels that compensate with longer minimum ROH thresholds systematically exclude shorter segments, yet our meta‐analytic results suggest that studies excluding shorter ROH segments may be underestimating the strength of inbreeding depression effects. This creates a contradiction in the literature where the studies most constrained by panel density are least likely to detect meaningful inbreeding depression signals. Standardization of ROH detection parameters, particularly minimum SNP density requirements, is therefore a pressing methodological priority for the field (Ferenčaković et al. 2013; Lavanchy and Goudet 2023).

Our meta‐analysis revealed that, when looking at moderators separately, survival and reproduction showed the strongest inbreeding depression effects (Figure 3), which is intuitive given that these traits are central to population fitness. However, trait type became non‐significant in our combined model, which also included variation in ROH length thresholds as a moderator. This may reflect collinearity between trait type and study system, given that wildlife studies disproportionately measure survival and reproduction due to their relevance to population persistence. Wildlife populations showed a consistently strong and robust signal for inbreeding depression across models, which aligns with theory predicting stronger inbreeding depression in smaller populations (Spigler et al. 2017). It is worth noting that wildlife studies tended to use denser SNP panels or WGS, and as our ROH threshold finding demonstrates, more comprehensive detection approaches are associated with stronger effect sizes. Methodological differences among study systems may therefore partially contribute to the observed wildlife‐human divergence, and this cannot be fully disentangled from biological differences in the current dataset. Nevertheless, we consider study system likely to reflect a genuine biological signal, consistent with theory and empirical evidence predicting stronger inbreeding depression in small, fragmented populations and ongoing habitat loss is intensifying these pressures (Fox and Reed 2011; Frankham et al. 2012). It is also possible that shared evolutionary history in the wildlife studies could influence the effect size patterns, especially given that the majority of studies are on mammalian taxa. More studies are needed in a wider range of wildlife species to fully understand the generality of our results, and whether certain taxonomic groups show stronger fitness effect sizes. Additionally, the measured effects of inbreeding are often strongest when fitness is measured over longer time scales, i.e., multiple life history stages or, for example, including lifetime reproductive success (Szulkin et al. 2007; Trask et al. 2021). While we have limited sample sizes, the two wildlife studies with lifetime reproductive success measurements had two of the strongest effect sizes (Data S2). More studies with fitness data encompassing multiple life stages or lifetime success may therefore strengthen the effect size of ROH on fitness declines.

Our meta‐analysis also uncovered a novel insight into the effect of ROH length on the detection and strength of inbreeding depression across populations. The finding that studies focusing exclusively on ROH ≥ 1 Mb detected a weaker inbreeding depression signal suggests that cumulative genetic load across the genome, including shorter segments reflecting distant inbreeding, matters more than considering only long ROH or recent inbreeding. Short ROH reflect ancestral inbreeding and the accumulation of deleterious recessive alleles over many generations, which can continue to present significant inbreeding depression in contemporary populations (Ceballos et al. 2018). This does not diminish the importance of long ROH, capturing recent or ongoing inbreeding, shown in many studies to predict strong negative fitness outcomes (Kardos et al. 2023; Stoffel et al. 2021b). It may be that long ROH have stronger effects on inbreeding than short ROH, but short ROH do also contribute. A better explanation of overall fitness loss is thus uncovered when including both together. It is worth noting, however, that this finding is based on a relatively small sample size and more studies will be needed to validate this finding.

Some studies reported that effect sizes were smaller than expected despite the presence of long ROH, attributing this to the purging of recessive deleterious alleles over generations of small population size (e.g., Sumreddee et al. 2019). Purging can reduce the frequency of strongly deleterious recessive variants, potentially attenuating inbreeding depression. However, purging cannot be relied upon as a management buffer. We find here that strong inbreeding depression can persist despite long histories of small effective population size (Kardos et al. 2023; Niskanen et al. 2020), indicating that mildly deleterious alleles, which are more resistant to purging, continue to accumulate and contribute to fitness declines.

The pathways through which genomic inbreeding affects fitness are varied and not yet fully understood. Inbreeding depression has been documented across multiple biological pathways, including reduced survival and longevity, impaired reproductive success, increased disease susceptibility and decreased immune function (Alshanbari and Aloraini 2025; Foster et al. 2026; Hasik et al. 2025; Niskanen et al. 2020; Swinford 2023). Disaggregating F_ROH_ by individual class and life stage reveals additional complexity, as maternal inbreeding can reduce offspring fitness independently of offspring inbreeding status (Bérénos et al. 2016; Stoffel et al. 2021a), suggesting that inbreeding effects cascade across generations in ways that aggregate F_ROH_ estimates may fail to capture. Population‐wide ROH frequency patterns also influence fitness interpretation: regions that are homozygous across many individuals simultaneously (ROH islands) are more likely to reflect positive selection than inbreeding load, while fitness costs have been primarily attributed to genome‐wide accumulation of ROH in non‐island regions in some studies (Stoffel et al. 2021a). These findings highlight that F_ROH_ disaggregation remains an underexplored but promising avenue for understanding when, and through what mechanisms inbreeding depression manifests, particularly in wild populations where early‐life and maternal effects may carry disproportionate consequences for population viability.

Overall, our findings confirm that genomic inbreeding reliably predicts fitness declines across taxa, with wildlife populations showing the strongest and most conservation‐relevant effects (Figure 2). Detecting these effects requires comprehensive ROH detection approaches that capture both recent and ancestral inbreeding, rather than restricting analyses to long ROH segments alone. Methodological standardization, including the use of higher‐density SNP panels and detection parameters set to include segments below 1 Mb, is a pressing priority for the field. Identifying ROH islands through population‐level frequency mapping should also be incorporated into analyses to distinguish inbreeding load from signatures of positive selection. Future work should prioritize stratification of F_ROH_ estimates by individual class and life stage in wild populations, where maternal and early‐life inbreeding effects may carry disproportionate consequences for population viability (Stoffel et al. 2021a). Together, these advances will strengthen the genomic toolkit available for assessing inbreeding risk and informing conservation management of small and isolated populations.

## Author Contributions

Julia C. Clarke, Rebecca S. Taylor and Micheline Manseau conceived the study. Julia C. Clarke performed data extraction and meta‐analysis and wrote the study. Rebecca S. Taylor and Micheline Manseau edited the study.

## Funding

This work was supported by the Government of Canada's Genomics Research and Development Initiative.

## Ethics Statement

The authors have nothing to report.

## Conflicts of Interest

The authors declare no conflicts of interest.

## Supporting information


**Table S1:** Search strategy used for systematic literature review (Web of Science and Google Scholar).
**Table S2:** Keyword inventory guiding search strategy development and screening.
**Figure S1:** PRISMA flow diagram.
**Figure S2:** The results of a random‐effects meta‐analysis investigating the relationship between genomic inbreeding (F_ROH_) and fitness across studies. The study ID links to the results in Data S1 and S2 bases for each paper included in the analysis. The effect size estimate and the 95% confidence intervals are shown for each dataset.
**Figure S3:** Egger's regression test to investigate funnel plot asymmetry due to potential publication bias.

**Data S1:** Spreadsheet 1 **|** The genomic data including details of all 44 studies included in the systematic literature review.

**Data S2:** Spreadsheet 2 **|** Details of the final 62 fitness effect sizes used in the meta‐analysis, as well as the details of all fitness effects from all the screened‐in studies.

## Data Availability

Extracted data for the review and meta‐analysis are available in Data S1 and S2. The ‘genomic data’ file includes details of all 44 studies included in the systematic literature review and the ‘fitness effects’ file includes the details of the final 62 effect sizes used in the meta‐analysis, as well as the details of all fitness effects from all of the screened in studies. All code used in the meta‐analysis is available on GitHub (https://github.com/BeckySTaylor/ROH‐meta‐analysis).
