# Supplementary material for: Runs of Homozygosity Predict Inbreeding Depression Across Taxa: A Systematic Review and Meta‐Analysis
Source: Mol Ecol. 2026 Sep 1;35(17):e70541. doi: 10.1111/mec.70541 (PMC13531397; doi:10.1111/mec.70541)
Supplement: Supplementary file 1 [file MEC-35-e70541-s001.docx]

**Supplemental Information for:**

**Runs of homozygosity predict inbreeding depression across taxa: a systematic review and meta-analysis**

Julia C. Clarke, Rebecca S. Taylor, and Micheline Manseau

**Table of Contents:**

| **Table S1** | Page 2 |
| --- | --- |
| **Table S2** | Page 3 |
| **Figure S1** | Page 4 |
| **Figure S2** | Page 5 |
| **Figure S3** | Page 6 |

# Table S1 | Search strategy used for systematic literature review (Web of Science and Google Scholar)

| **Database** | **Search String** | **Date Searched** | **Publication Date Range** | **Results (n)** |
| --- | --- | --- | --- | --- |
| Web of Science & Google Scholar | **Search String 1 (focused):** (Inbreeding OR "inbreeding depression") AND ("Runs of homozygosity" OR ROH) AND ("genomic inbreeding" OR "Identity by descent" OR IBD) AND Fitness | November 3–7, 2025 | 2000–present^[1]^ | 445 |
| Web of Science & Google Scholar | **Search String 2 (broad):** ("runs of homozygosity" OR ROH) AND (inbreeding OR "inbreeding depression" OR autozygosity) AND (Fitness) | November 3–7, 2025 | 2000–present^[2]^ | 2,900 |
| **Total identified** |  |  |  | **~3,345** |
| **After deduplication and removal of irrelevant records (incl. IBD/irritable bowel false positives)** |  |  |  | **325** |

# Table S2. Keyword inventory guiding search strategy development and screening

| **Category** | **Keywords** |
| --- | --- |
| Primary | Inbreeding, inbreeding depression, ROH, runs of homozygosity, F_ROH, genomic inbreeding, identity by descent, IBD, autozygosity, homozygosity |
| Methodological | Whole genome sequencing, SNP sequencing, population genomics, population genetics |
| Outcome | Fitness, fitness effects, survival, conservation |

*Note: Related terms and synonyms were considered during title/abstract screening to ensure comprehensive capture beyond the listed terms.*

**Figure S1** | PRISMA flow diagram

**Figure S2** | The results of a random-effects meta-analysis investigating the relationship between genomic inbreeding (F_ROH_) and fitness across studies. The study ID links to the results in the Supplementary databases for each paper included in the analysis. The effect size estimate and the 95% confidence intervals are shown for each dataset.

**FIGURE S3** | Egger’s regression test to investigate funnel plot asymmetry due to potential publication bias.

1. The 2000–present range was selected because ROH-based studies became widely available after this date; foundational pre-2000 papers already known to the reviewer were included regardless of date.
